# Digital Technology Use Among Individuals with Schizophrenia: Results of an Online Survey

**DOI:** 10.2196/mental.5379

**Published:** 2016-05-04

**Authors:** Katrina Gay, John Torous, Adam Joseph, Anand Pandya, Ken Duckworth

**Affiliations:** ^1^ NAMI, the National Alliance on Mental Illness Washington DC, DC United States; ^2^ Department of Psychiatry Beth Israel Deaconess Medical Center Harvard Medical School Boston, MA United States; ^3^ Fulbright-Nehru Scholar Boston, MA United States; ^4^ Department of Psychiatry University of Southern California Health Sciences Los Angeles, CA United States; ^5^ Department of Psychiatry Harvard Medical School Boston, MA United States

**Keywords:** Schizophrenia, Computers, Technology, Internet, Coping, Recovery

## Abstract

**Background:**

Despite growing interest in the use of digital technology by individuals with schizophrenia, little is known about how these individual relate to, own, and use technology in their daily life and in the context of their symptoms.

**Objective:**

The goal of this study is to better characterize technology use in those with schizophrenia.

**Methods:**

A Web-based survey of individuals’ use of and attitudes toward technology for those 18 years and older self-identifying as having schizophrenia, schizoaffective disorder, or schizophrenia spectrum disorders was conducted. Consumer input was sought in the design of the survey.

**Results:**

In total, 457 individuals responded to this Web-based survey. Ninety percent owned more than one device (personal computer, landline telephone, tablet, public computer, mobile phone without applications or Internet, or smartphone), with many reporting high utilization of multiple devices, and 61% having 2 devices. The respondents reported that Web-based technology helped with support from family and friends, as well as in gathering information. Many respondents used Web-based technology to help identify coping strategies (24% very often or often) including music to help block or manage voices (42%), while others used technology to set alarms/reminders for medication management (28%). Younger respondents in particular anticipated the role of technology growing over time with respect to their recovery.

**Conclusions:**

Survey respondents reported that technology access was common, with utilization involving coping, reminders for medications and appointments, and connection. Overall, attitudes were largely positive. Overuse was a concern for 30% of respondents. The study is limited in its generalizability as the population was highly engaged in mental health treatment (87%), self-identified as living with the disorder, and had awareness of their illness. This survey demonstrates high engagement for a subset of technology-oriented individuals living with schizophrenia. It is not known what percent of individuals with schizophrenia are represented by these technology-oriented survey respondents.

## Introduction

Mobile health (mHealth) has emerged as a movement to harness connected, digital tools such as computers, tablets, mobile phones, and wearables with an aim to advance health care. Such digital devices have the potential to decrease health care costs, increase access to care, and offer novel diagnostic, monitoring, and treatment options across a broad range of diseases [1].

Mental illness is a crucial target for mHealth. mHealth can directly address a number of the devastating characteristics of mental illness, including its chronic nature, stigma, dynamic symptoms, and lack of easy access to treatment. In addition, mHealth can assist individuals in managing their conditions and help empower them to be active participants in their own recovery, a key to improved outcomes.

Schizophrenia is a mental illness for which mHealth offers a tremendous opportunity to deliver personalized, innovative, and accessible solutions. Schizophrenia is a health condition impacting approximately 1% of the population worldwide, afflicting men and woman with equal prevalence, often beginning in the late teens or twenties, and frequently characterized by chronic symptoms including delusions, hallucinations, and disorganization [2]. While outcomes can be poor for some [3], with early interventions [4], psychosocial support [5], judicious medication management [6], and appropriate medical care [7], individuals with schizophrenia can lead rich and fulfilling lives.

However, individuals with schizophrenia remain at risk of relapse, which can be difficult to predict, may struggle with more difficulty accessing appropriate care than others with chronic conditions, and often face tremendous social and emotional obstacles in their recovery [8]. Since their inception, digital technologies have been explored as tools to offer better mental health care. Twenty-three years ago, researchers were using the first mobile phones to help patients with anxiety disorders [9]. With the rise of mobile handheld technologies such as personal digital assistants, clinical studies suggested that those with serious mental illness were able to use and adhere to mobile interventions [10], even when experiencing negative symptoms [11], and that they generally found such technology helpful and easy to use. However, the advantages of such early mobile technological solutions involved practical limitations such as high cost and limited ownership.

However, the situation has changed in the last several years, as connected and mobile technologies are becoming more prevalent and affordable. Along with the rest of the population, those with schizophrenia are increasingly owning mobile technological devices such as mobile phones [12] and are using them to digitally connect. A recent meta-analysis of mobile phone ownership among those with symptoms of psychosis revealed that the rate of phone ownership was rapidly increasing, with 81.4% ownership among those surveyed in 2014 and 2015 [13]. Individuals with schizophrenia not only own connected devices, but are also able to use them for their mental health care. A recent systematic review of mobile phone studies and schizophrenia found no evidence of any adverse events related to technology use and rather overall strong support, interest, and adherence among those with schizophrenia [14]. There is also emerging data on how those with schizophrenia engage with digital technology; one recent study of 80 individuals reports that just over half of respondents used text messaging (short message service, SMS), nearly half had email accounts, and over a quarter used social networking websites [15].

Yet, despite the increasing potential of digital technologies for the treatment of schizophrenia, little is known about how individuals with schizophrenia use, interact with, and feel about these mobile health tools. While previous studies have examined serious mental illness and technology [16,17], less is known about how people with schizophrenia actually use connected devices. Just as “early adopters” of consumer electronics are an important segment in market research, important lessons may be learned about technology use in schizophrenia from even the more engaged individuals. mHealth technologies now offer the potential for advanced personalized care; however, that potential starts with understanding how people are actually using these technologies. In this paper, we present results from a Web-based survey on mobile technology that captures a subset of those with schizophrenia who are more engaged with technology.

## Methods

In 2014, the National Alliance on Mental Illness (NAMI) commissioned Harris Poll to conduct a survey on technology use among those self-identifying as having schizophrenia. Harris Poll is an international market research firm, which in part specializes in Internet-based polls. Harris Poll conducted 457 interviews via a Web-based survey that averaged 15 minutes in length from August 25 to September 8, 2014.

The survey was developed and distributed to assess the role and use of technology among a subset of individuals who self-identified as having schizophrenia, schizoaffective disorder, and schizophrenia spectrum disorder.

The survey was designed to elicit individual responses to questions about technology use. Survey questions focused on access to digital devices, frequency of use, purposes of use including coping strategies, experience of technology use including risks of overuse, and perceptions of the possible role of technology in the future. Inclusion criteria were the following: 18 years or older, living in the United States, and self-reporting having been diagnosed with schizophrenia, schizoaffective disorder, or another schizophrenia spectrum disorder. Subjects were recruited from a sample obtained by Harris Poll (65%), NAMI’s mailing list (15%), and NAMI’s website (20%).

To help adjust for attitudinal and behavioral differences between those who use the Internet versus those who do not, those who join Web-based panels versus those who do not, and those who responded to this survey versus those who did not, results were weighted using a propensity score. This propensity weighting is proprietary to Harris Poll and its parent company, Nielsen, and used frequently in their international survey work. This propensity score, used to minimize the sociodemographic, attitudinal, and behavioral differences between Web-based and phone respondents, was calculated using a logistic regression model, based on the theory of individual choice. This weighted data set was then used to create a profile for those meeting the full qualification criteria, and was applied to the entire sample using the attitudinal and behavioral variables listed above. The weight also included a variable to account for the frequency of visiting the NAMI website [18]. Both Harris Poll and NAMI analyzed the data. Of note, results presented in this paper are based on this weighting score, although NAMI will offer the raw data to mental health researchers upon request. This study was approved by the University of Southern California Institutional Review Board.

## Results

A complete copy of the survey is accessible at the NAMI website [19]. These results are weighted as explained in the methods section

### Survey Respondents

As indicated in Table 1 , 457 subjects completed the survey. Thirty-nine percent of subjects (179/457) were aged 18-34, 23% (107/457) were aged 35-46, 30% (136/457) were aged 47-64, and 8% (35/457) were older than 65. The median age of respondents was 41.3 years and 46% were female. Seventy-one percent of respondents were white and 48% reported a household income less than $24,999. The mean age of onset of schizophrenia in respondents was 25.5 years. Eighty-seven percent of respondents were in current treatment for schizophrenia. Details of demographics are shown below in Table 1.

**Table 1:** Demographics

Variable		n (%) or mean (SD)
**Gender (n=457)**	Male	247 (54)
	Female	210 (46)
**Age (n=457)**	18-34	179 (39)
	35-46	107 (23)
	47-64	136 (30)
	65+	35 (8)
	Mean	41.3 (13.96)
**Ethnicity (n=457)**	White	324 (71)
	Hispanic	32 (7)
	Black/African American	64 (14)
	Asian or Pacific Islander	14 (3)
	Native American or Alaskan Native	5 (1)
	Mixed race	1 (0)
	Other race	5 (1)
	Declined to answer	14 (3)
**Highest Level of Education (n=457)**	High school or less	165 (36)
	Job specific training program(s) after high school	50 (11)
	Attended college or college degree	215 (47)
	Attended graduate school or graduate degree	27 (6)
**Household Income (n=457)**	Less than $24,999	219 (48)
	$25,000 - $49,999	91 (20)
	$50,000 - $99,999	77 (17)
	$100,000 or more	41 (9)
	Declined to answer	31 (7)
**Employment Status (n=457)**	Full time	86 (19)
	Part time	55 (12)
	Self-employed	18 (4)
	Not employed, but looking for work	32 (7)
	Not employed and not looking for work	18 (4)
	Not employed, unable to work due to a disability or illness	151 (33)
	Retired	41 (9)
	Stay-at-home spouse or partner	32 (7)
	Student	23 (5)
**Health Insurance Status (n=457)**	Covered	411 (90)
	Not covered	46 (10)
**Type of Health Insurance Among Those Covered (n=415)**	Health insurance or HMO^a^through work or union or someone else’s work or union	54 (13)
	Health insurance or HMO bought directly by me or another member of my family	50 (12)
	Medicare or a Medicare HMO	158 (38)
	Medicaid, Medicaid HMO, or medical assistance	133 (32)
	Health insurance from another source	20 (5)
**Region (n=457)**	East	101 (22)
	Midwest	118 (26)
	South	137 (30)
	West	101 (22)

^a^Health Maintenance Organization

### Access to Digital Devices

When asked about access to personal computers, smartphones, landline phones, tablets, public computers, and mobile phones without Internet capabilities, 90% (411/457) had access to more than one of the items listed, 61% (279/457) had access to 2 or 3 devices, and 29% (133/457) had access to 4 or more. Eighty-nine percent (407/457) had access to a personal computer, 54% (247/457) to a smartphone, 52% (238/457) to a landline, 35% (160/457) to a tablet, 32% (146/457) to a public computer, and 31% (141/457) to a mobile phone without applications or Internet accessibility. Access to technology also varied by age with 68% (122/179) of those aged 18-34, 48% (52/107) of those aged 35-46, 44% (59/136) of those aged 47-64, and 37% (13/35) of those older than 65 possessing a smartphone.

### Frequency of Use

In examining time spent on devices per day, results indicated that 89% (407/457) of those living with schizophrenia spent one or more hours per day on their personal computer, and 18% (82/457) spent 10 or more hours per day. For mobile phones, results suggested that 85% (388/457) spent one or more hours per day on the device, while nearly half, 48% (219/457), spent 3 or more hours per day. Sixty-six (302/457) percent of respondents anticipated that technology would become a bigger part of their recovery in the coming years.

### Purpose of Use

The most common activities during device use were surfing the Internet (2.7 hours), visiting social networking sites (2.0 hours), playing Web-based games (1.4 hours), and sending text messages (1.3 hours). Thirty-six percent (165/457) reported using Web-based technology to cope with schizophrenia “often” or “very often.” Twenty-four percent (110/457) reported using technology “sometimes,” and 40% (183/457) reported using technology “rarely” or “never” to cope with their illness.

Respondents reported using technology to cope with their illness “often” or “very often” with respect to music or audio files to block or manage auditory hallucinations (42%, 192/457); information about mental health on the Internet (38%, 174/457); calendar reminders for appointments or setting alarms (37%, 169/457); transportation, GPS, and map needs (32%, 146/457); medication management (28%, 128/457); providing support for others (26%, 119/457); developing relationships with others who have lived experience of schizophrenia (26%, 119/457); monitoring symptoms (25%, 114/457); and identifying coping strategies (24%, 110/457). Response rates varied by age: 34% (61/179) of those aged 18-34, 24% aged 35-46 (26/107), 14% aged 47-64 (19/136), and 8% of those aged 65 or older (3/35) reported that they would use various technologies for coping with symptoms of schizophrenia.

Twenty-three percent of respondents (150/457) “often” or “very often” avoided offline activities in order to stay on the Internet, and 18% (82/457) reported “often” or “very often” neglecting responsibilities because of Internet use.

For those who reported having the following relationships, respondents used their devices to communicate “often” or “very often” with family (51%, 233/457) and friends (48%, 219/457) and least often with case managers (23%, 105/457), professors (22%, 101/457), their doctors (22%, 101/457), or peer supporters (22%, 101/457).

### Experiences of Using Digital Devices

Respondents rated the helpfulness of various activities that they engaged in via digital devices. Surfing the Internet was reported to be the most helpful activity for 42% (192/457) of respondents, followed by talking on a landline, mobile phone, or smartphone (39%, 178/457), sending personal emails (31%, 142/457), text messaging (31%, 142/457), spending time on social networking sites (29%, 133/457), online gaming (26%, 119/457), and participating in online chat rooms or discussion groups (21%, 96/457).

When asked about computer, tablet, or mobile phone use, survey respondents were more likely to report positive feelings about their device usage (75%, 343/457). Participants felt connected (58%, 265/457), happy (47%, 215/457), inspired (47%, 215/457), hopeful (45%, 206/457), peaceful (44%, 201/457), motivated (43%, 197/457), and empowered (33%, 151/457), “often” or “very often” during their digital device usage.

Negative feelings were reported “often” or “very often” 56% (255/457) of the time, including feelings of being unable to stop (27%, 123/457), frustration (25%, 114/457), paranoia (24%, 110/457), worry (20%, 91/457), sadness (20%, 91/457), anger (19%, 87/457), mania (16%, 73/457), or envy (16%, 73/457).

Respondents also indicated that they were more likely to use technology when feeling well (58%, 265/457, reported “often” or “very often”) as compared to when they were experiencing many symptoms (30%, 137/457). Respondents indicated that surfing the Internet, talking to others on the phone, spending time on social networking sites, and sending emails and text messages were helpful activities in managing their illness. To better understand how views differed between those who had versus had not used technology, we compared how nonusers versus users rated the helpfulness of various technologies. In Table 2 , “perceived” helpfulness refers to the views of those who had not used that technology and “actual” to the views of those who had used it.

**Table 2:** Mean helpfulness ratings (actual vs. perceived) of activities on a scale from 1 to 10, where 10 was the most helpful and 1 was the least helpful.

	Actual	Perceived
Surfing the Internet	6.1	4.2
Talking on the telephone including on a landline, mobile phone, or smartphone	5.8	3.7
Using social networking sites	5.3	2.4
Text messaging	5.3	2.8
Sending personal emails	5.1	3.3
Joining or participating in online chat rooms or discussion groups	5	3.3
Online gaming	5	2.7
Video chatting	4.7	2.9

## Discussion

### Principal Findings

This is the largest study to date examining ownership and use of technology among those with schizophrenia. While conducting this study via a Web-based survey created a response bias (respondents are likely to be more technology savvy), the results offer crucial insights into how those with schizophrenia engage and connect with technology, suggesting potential targets for further study and possible considerations for clinical care.

### Access to Digital Devices and Frequency of Use

As expected in a Web-based survey, *access* to connected devices was high in the surveyed population—although results were similar to early smaller studies. This study demonstrates that many individuals living with schizophrenia have access to connected devices with results suggesting that the majority (61%, 279/457) actually have access to two or three devices. Rates of access to technology in this survey sample were similar to such rates in the general population, with 54% (247/457) of respondents having access to a smartphone compared to 64% of Americans currently owning one [20]. Although 54% is also similar to the rate of ownership of 58% reported in a survey study at a state clinic treating those with serious mental illness [12], it is higher than the rate of ownership of 37% reported in another recent survey study of those with serious mental illness at a community health center [21]. The fact that this study was not limited to a single clinic and was conducted as an Internet survey may explain the higher ownership rates reported here. Results also showed that ownership of technology was biased toward younger individuals with schizophrenia, which is in line with national general population trends [20] and earlier survey studies on technology use in patients with mental illness [13,22]. This suggests that technology use in clinical care may be more fruitful when targeted toward younger individuals, such as those with prodromal or first episode symptoms; recent survey research supports this population’s strong interest in technology as part of their care [23].

Survey results also indicated that digital device utilization among people with schizophrenia as similar in manner to that of the general population—individuals with schizophrenia spent the most time with their connected devices talking to others, followed by surfing the Internet, browsing social networking sites, gaming, and text messaging. A recent Pew survey of the general population showed that the four most common uses of mobile phones were texting, voice calls, Internet browsing, and text messaging [20].

Individuals living with schizophrenia may face a double stigma when using digital devices. Beyond the stigma often associated with schizophrenia itself [24], there may be bias that those living with schizophrenia do not own, cannot use, are not interested in, or will become more paranoid and agitated when using technologies like mobile phones [25]. Our survey indicates that there is a subset of individuals with schizophrenia who challenge the assumptions of low access and low frequency of use. While our methodology of a Web-based survey excludes many who do not like or use technology, our results highlight a subset of those with schizophrenia who not only are well connected, but also use technology in a similar manner to the general population.

The nature of this survey administered on the Internet also allowed us to capture information on those who may be too connected. Results revealed that 18% (82/457) of respondents reported using their personal computer and 14% (64/457) reported using their mobile phone for 10 hours or more per day. Therefore, rather than being “underconnected,” some in this sample of individuals living with schizophrenia may have overused digital devices. Twenty-three percent (105/457) of respondents “often” or “very often” avoided activities to stay on the Internet, and 18% (82/457) neglected responsibilities because of the Internet. Future work will need to examine the causes and effects of digital device overuse in populations with schizophrenia; exploring how they use the Internet has the potential to reveal important insights. While we often consider those who are not connected and not using technology, ensuring that we also understand those who may be overconnected is also important, especially given how little we know about the impact of excessive digital device use on the symptoms and course of schizophrenia.

### Purpose of Use and Coping Strategies

**Figure 1:**
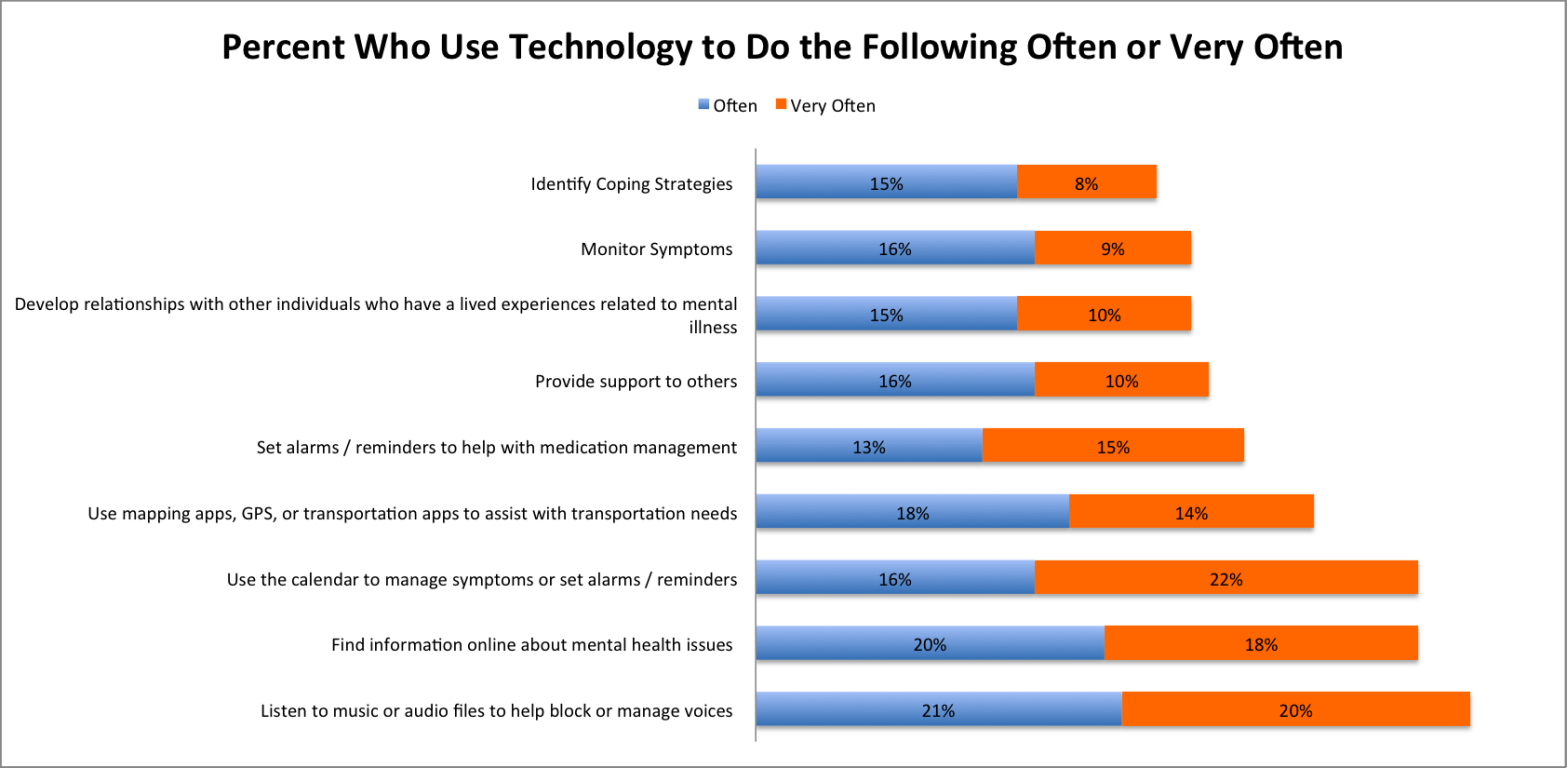
Reported use of technology in coping based on 457 responses to Question 4: Aside from telephone calls, how frequently do you use a computer, tablet, or cell/smartphone to do the following?

As shown in Figure 1 , the survey results suggest that many people with schizophrenia already use their mobile phones to manage their illness and promote their recovery. However, nearly half (48%, 219/457) of respondents reported that they “rarely” or “never” used their devices to communicate with others when experiencing symptoms, raising concerns about the ability of various technology interventions to deliver assistance when it may potentially be helpful. Results indicating that the highest use of technology for coping was among younger individuals suggests that such tools may be best accepted among a younger population; however, those who are older should not be excluded from use. Finally, considering again the bias of our survey toward more connected individuals, the reported reluctance to use technology when in crisis is especially concerning and underscores the importance of not overrelying on technology for crisis planning and intervention.

### Experiences of Using Digital Devices

While positive feelings toward mobile devices were more common, our study results indicate that individuals with schizophrenia have both positive and negative feelings toward their mobile devices. However, these results are not unique to individuals with schizophrenia: 77% of the general population reported that their mobile phones made them feel “happy,” 57% reported they felt “distracted, 36% “frustrated,” and 15% “angry” because of their devices [17]. Of the survey respondents with schizophrenia, 25% (114/457) reported feeling “frustrated,” 24% (110/457) “paranoid,” and 19% (87/457) “angry,” suggesting that the feelings caused by use of technology are similar between the general population and those living with schizophrenia. The lack of any strong signal regarding negative experiences with technology is also in line with a recent systematic review of the literature on mobile phone interventions in those with schizophrenia, which also found no evidence of adverse events such as increased paranoia, fear, or anger [13]. However, some who are afraid of technology may not have taken this Web-based survey, so it is difficult to generalize these results.

### Limitations

While this is the largest study to date examining technology use specifically in people living with schizophrenia, several serious limitations exist. Because this was a Web-based survey, there was a strong sampling bias toward those who had access to the Internet. The respondents of the survey represented a population that was highly engaged in treatment (87%, 398/457), which is above the average nationally. In addition, the survey respondents were not representative of the demographic distribution of individuals living with schizophrenia, since Caucasians were overrepresented in our sample (71%, 325/457). Furthermore, the majority of survey respondents were younger; due to the low sample size of those over age 65, the results for older people with schizophrenia must be interpreted with caution. This survey may be considered to have included a subpopulation of individuals who self-identified as having schizophrenia, were engaged in treatment at a high level, and were technology oriented. These results cannot, therefore, be generalized to the broader population of individuals with schizophrenia; however, they do still represent a segment of those engaged in their recovery and treatment. The fact that our results, especially in regards to device ownership, were similar to prior surveys of technology use in schizophrenia is encouraging. While the study design did not allow us to ascertain if technology use itself was correlated with higher rates of treatment adherence or general functioning, such an analysis is possible and will be an important topic for future research. Our finding that at least some individuals with schizophrenia are well connected digitally underscores the importance of conducting further studies to examine those who are not as connected and why such differences exist. While our survey focused on those who may be more connected, the results are also important in countering assumptions and stigma around those with mental illness and schizophrenia.

### Conclusion

There is a subset of individuals living with schizophrenia who often have access to several mobile and Internet-connected devices such as mobile phones and personal computers. They use them in a similar manner as the general population: to make phone calls, browse the Internet, and send text messages. Our results suggest that they use technology in positive ways—for coping, appointments and medication reminders, and connection to family, friends, and peers. For some there is the suggestion of a risk of overuse, as in the general population. While our survey indicates that there is a digitally engaged subset of those with schizophrenia, it does not tell us how we can best leverage technology to improve outcomes, or suggest methods to reach less-connected individuals to better utilize technology to improve outcomes. What our survey does tell us is that these are important questions to be explored, and that we can realize and leverage mHealth solutions in schizophrenia.

As technology continues to be a growing force in modern life and in health care practice, our results demonstrate that opportunities exist to further engage some individuals with schizophrenia in support of their recovery. While our survey does not inform us about those who are not connected and online, understanding those who are connected underscores that schizophrenia should not be considered a barrier to mHealth innovation and the use of digital health tools. Our survey also suggests that technology alone is not a panacea and that there are potential risks associated with technology use. As technology and mHealth continue to expand, it is critical we that we study and understand how mobile devices and Internet tools can be used to further promote recovery in individuals living with schizophrenia.

## Abbreviations

mHealth: mobile health

## References

[1] Silva BM, Rodrigues JJ, de la Torre Díez I, López-Coronado M, Saleem K (2015). Mobile-health: A review of current state in 2015. J Biomed Inform.

[2] Tandon R, Keshavan MS, Nasrallah HA (2008). Schizophrenia, "Just the Facts": What we know in 2008 part 1: Overview. Schizophr Res.

[3] Zipursky RB (2014). Why are the outcomes in patients with schizophrenia so poor?. J Clin Psychiatry.

[4] Mokhtari M, Rajarethinam R (2013). Early intervention and the treatment of prodrome in schizophrenia: A review of recent developments. J Psychiatr Pract.

[5] Chien WT, Leung SF, Yeung FK, Wong WK (2013). Current approaches to treatments for schizophrenia spectrum disorders, part II: Psychosocial interventions and patient-focused perspectives in psychiatric care. Neuropsychiatr Dis Treat.

[6] Mokhtari M, Rajarethinam R (2013). Early intervention and the treatment of prodrome in schizophrenia: A review of recent developments. J Psychiatr Pract.

[7] Mokhtari M, Rajarethinam R (2013). Early intervention and the treatment of prodrome in schizophrenia: A review of recent developments. J Psychiatr Pract.

[8] Pandya A, Bresee C, Duckworth K, Gay K, Fitzpatrick M (2011). Perceived impact of the disclosure of a schizophrenia diagnosis. Community Ment Health J.

[9] Pandya A, Bresee C, Duckworth K, Gay K, Fitzpatrick M (2011). Perceived impact of the disclosure of a schizophrenia diagnosis. Community Ment Health J.

[10] Depp CA, Mausbach B, Granholm E, Cardenas V, Ben-Zeev D, Patterson TL, Lebowitz BD, Jeste DV (2010). Mobile interventions for severe mental illness: Design and preliminary data from three approaches. J Nerv Ment Dis.

[11] Kimhy D, Corcoran C (2008). Use of Palm computer as an adjunct to cognitive-behavioural therapy with an ultra-high-risk patient: A case report. Early Interv Psychiatry.

[12] Torous J, Chan SR, Yee-Marie TS, Behrens J, Mathew I, Conrad EJ, Hinton L, Yellowlees P, Keshavan M (2014). Patient smartphone ownership and interest in mobile apps to monitor symptoms of mental health conditions: A survey in four geographically distinct psychiatric clinics. JMIR Ment Health.

[13] Firth J, Torous J (2015). Smartphone apps for schizophrenia: A systematic review. JMIR Mhealth Uhealth.

[14] Firth J, Cotter J, Torous J, Bucci S, Firth JA, Yung AR (2015). Mobile phone ownership and endorsement of "mHealth" among people with psychosis: A meta-analysis of cross-sectional studies. Schizophr Bull.

[15] Miller BJ, Stewart A, Schrimsher J, Peeples D, Buckley PF (2015). How connected are people with schizophrenia? Cell phone, computer, email, and social media use. Psychiatry Res.

[16] Alvarez-Jimenez M, Alcazar-Corcoles MA, González-Blanch C, Bendall S, McGorry PD, Gleeson JF (2014). Online, social media and mobile technologies for psychosis treatment: A systematic review on novel user-led interventions. Schizophr Res.

[17] Ben-Zeev D, Brenner CJ, Begale M, Duffecy J, Mohr DC, Mueser KT (2014). Feasibility, acceptability, and preliminary efficacy of a smartphone intervention for schizophrenia. Schizophr Bull.

[18] National Alliance on Mental Illness.

[19] (2014). National Alliance on Mental Illness.

[20] Smith A (2015). Pew Research Center.

[21] Glick G, Druss B, Pina J, Lally C, Conde M (2015). Use of mobile technology in a community mental health setting. J Telemed Telecare.

[22] Torous J, Friedman R, Keshavan M (2014). Smartphone ownership and interest in mobile applications to monitor symptoms of mental health conditions. JMIR Mhealth Uhealth.

[23] Lal S, Dell'Elce J, Tucci N, Fuhrer R, Tamblyn R, Malla A (2015). Preferences of young adults with first-episode psychosis for receiving specialized mental health services using technology: A survey study. JMIR Ment Health.

[24] Allerby K, Sameby B, Brain C, Joas E, Quinlan P, Sjöström N, Burns T, Waern M (2015). Stigma and burden among relatives of persons with schizophrenia: Results from the Swedish COAST Study. Psychiatr Serv.

[25] Ennis L, Rose D, Denis M, Pandit N, Wykes T (2012). Can't surf, won't surf: The digital divide in mental health. J Ment Health.

